# Structural insights into urocanate reductase using room-temperature X-ray crystallography

**DOI:** 10.1107/S2059798326003360

**Published:** 2026-05-05

**Authors:** Swati Aggarwal, Nitisha Gurav, Esko Oksanen, Karin Lindkvist-Petersson, Raminta Venskutonytė

**Affiliations:** a European Spallation Source ERIC (ESS) 221 00Lund Sweden; b MAX IV Laboratory Lund University Fotongatan 2 224 84Lund Sweden; c LINXS – Institute of Advanced Neutron and X-ray Science, Lund, Sweden; d Department of Computational Chemistry Lund University 221 84Lund Sweden; e Department of Experimental Medical Science Lund University 221 84Lund Sweden; University of Western Australia, Crawley, Australia

**Keywords:** urocanate reductase, room-temperature crystallography, active-site anion interactions

## Abstract

Urocanate reductase (UrdA) is a bacterial enzyme that converts urocanic acid to imidazole propionate. Its catalytic residue Arg411 undergoes a large conformational change in the substrate-bound versus product-bound states. In contrast to previously studied cryo-conditions, the room-temperature X-ray data of UrdA presented here show that the occupancy distribution of Arg411 is affected by crystal cryocooling. We further provide evidence that a phosphate ion stabilizes the substrate complex and can bias the conformation of Arg411. Both room-temperature and cryogenic X-ray datasets were essential to elucidate the dynamic nature of the UrdA active site, highlighting the importance of collecting data at both temperatures, as each may reveal distinct conformational states.

## Abstract 2

The active-site dynamics of the microbial enzyme urocanate reductase were revealed by room-temperature X-ray crystallography.

## 1. Introduction

Urocanate reductase (UrdA) is a bacterial flavoenzyme which catalyses the conversion of urocanic acid to imidazole propionate (Bogachev *et al.*, 2012 ▸). The latter metabolite has been linked to a variety of human diseases. UrdA is expressed by bacteria present in the human gut, and research on its health effects in humans is gaining increased attention (Koh *et al.*, 2018 ▸; Mastrangelo *et al.*, 2025 ▸; Park *et al.*, 2025 ▸). We have previously determined high-resolution X-ray structures of UrdA from *Shewanella oneidensis* in four different structural states that provided high-resolution details of its active site and enzymatic mechanism (Venskutonytė *et al.*, 2021 ▸). These structures were achieved using cryo-crystallography, *i.e.* executing data collection at cryogenic (∼100 K) temperatures. Cryo-crystallography has been the standard in macromolecular crystallography since the 1990s due to its ability to mitigate radiation damage, extend crystal lifetimes and facilitate automated sample handling (Haas, 2020 ▸). However, at cryogenic temperatures water and solvent networks vitrify, reducing atomic mobility and dynamic disorder. While this stabilizes the lattice, it also forces proteins into a limited subset of conformations, potentially distorting functionally relevant ensembles (Fraser *et al.*, 2011 ▸). Comparative studies reveal that numerous side chains, loops and even bound ligands adopt alternative conformations at room temperature (RT) that are not observed under cryogenic conditions, which can in turn lead to incorrect mechanistic interpretations (Ebrahim *et al.*, 2022 ▸; Keedy *et al.*, 2014 ▸; Milano *et al.*, 2022 ▸). One such example is the enzyme dihydrofolate reductase, where RT datasets revealed functionally relevant heterogeneity that cryo-data masked or altered, emphasizing that cryocooling does not simply trap a subset of RT states, but can reshape the conformational landscape (Keedy *et al.*, 2014 ▸). Similar effects have been reported in ligand-binding proteins such as protein tyrosine phosphatase 1B (PTP1B), where RT structures expose alternate ligand poses and allosteric states that were hidden in models under cryo-conditions (Skaist Mehlman *et al.*, 2023 ▸).

Nonetheless, RT experiments face challenges, notably increased radiation sensitivity and crystal dehydration. Modern detectors, micro-focus beamlines and fast data-collection protocols now alleviate these issues, enabling the collection of complete single-crystal datasets before radiation damage accumulates (Doukov *et al.*, 2020 ▸). Although cryo data collection remains practical for routine high-throughput pipelines, the complementarity of RT data is increasingly recognized. Thus, combining both methods provides a more complete picture of macromolecular function, with RT datasets revealing the dynamic and physiological ensemble, while cryo datasets capture high-resolution static details.

In this study, we determined the structure of UrdA by RT X-ray crystallography. We have analyzed the substrate-bound (UrdA′S-RT) and product-bound (UrdA′P-RT) structures of the two-domain UrdA (UrdA′, consisting of a FAD-binding and a mobile clamp domain together forming an active site) in the presence and absence of sulfate ions. While our results corroborate the previously observed mechanism involving the catalytic Arg411 residue in the active site, we show that the conformational landscape of this residue is affected by crystal cryocooling. Arg411 samples two different conformations in the substrate-bound and product-bound states, respectively, and we observe a shift in occupancy distribution from the product-like state under cryo-conditions towards the substrate-like state at RT for the UrdA′P structure. Moreover, we show that Arg411 is stabilized by a sulfate or phosphate ion, which alters the conformation of this residue.

## 2. Materials and methods

### 2.1. UrdA′ crystallization

The truncated urocanate reductase (UrdA′) protein was expressed and purified as described previously (Venskutonytė *et al.*, 2021 ▸). UrdA′ was concentrated to around 20–30 mg ml^−1^ and was subjected to crystallization using the sitting-drop method in 24-well plates (Hampton Research, USA). UrdA′ in complex with substrate (UrdA′S-RT) and sulfate was obtained in a condition consisting of 20% PEG 8000, 0.3 *M* ammonium sulfate, 0.1 *M* HEPES pH 7.0 in a drop with equal volumes of protein and reservoir solution (1.1 + 1.1 µl); UrdA′ in complex with product (UrdA′P-RT) and sulfate was obtained under the same conditions but with 24% PEG 8000 and using a 1.5 + 1.5 µl drop. Prior to setting up the drops, the protein sample was supplemented with excess FAD (2.4–4.7 m*M*) and 2.5 m*M* urocanate or 4.7 m*M* imidazole propionate. The crystals used for data collection with ammonium citrate were obtained in a condition consisting of 20% PEG 8000, 0.2 *M* ammonium citrate, 0.1 *M* HEPES pH 7.0 in a drop consisting of 1.5 µl well solution and 1.5 µl reservoir solution. Prior to setting up the drops, the protein sample was supplemented with 2.4 m*M* FAD and 2.5 m*M* urocanate (UrdA′S) or 10 m*M* imidazole propionate (UrdA′P). The crystal of UrdA′ in complex with product (UrdA′P-cryo) and sulfate used for data collection under cryo-conditions was obtained using crystallization conditions consisting of 20% PEG 8000, 0.2 *M* ammonium sulfate, 0.1 *M* HEPES pH 7.0 by the hanging-drop vapor-diffusion method, mixing 1 µl protein solution with 1 µl reservoir solution and immersing into reservoir solution supplemented with 20% glycerol as a cryoprotectant prior to flash-cooling in liquid nitrogen.

### 2.2. X-ray data collection and structure determination

Each dataset presented in the study was collected from a single crystal. All room-temperature datasets were collected on the BioMAX beamline at MAX IV, Lund, Sweden (Ursby *et al.*, 2020 ▸; Gonzalez *et al.*, 2025 ▸). For RT collection, the crystals were harvested using MiTeGen dual-thickness microloops (SKU-MS-L18SP-200), which were inserted into MicroRT polyester capillaries (MiTeGen, USA) prefilled with 20 µl reservoir solution and sealed around the base of the loop using vacuum grease. The crystal was then immediately mounted on the goniometer and diffraction data were collected. The UrdA′P-cryo dataset was collected on beamline P14 EMBL at DESY, Hamburg, Germany. The UrdA′P-cryo crystal data with sulfate and the UrdA′P-RT crystal data with citrate were processed using *XDS* (Kabsch, 2010 ▸), and for the UrdA′S-RT dataset with citrate and the UrdA′S-RT dataset with sulfate *EDNA*2_*proc* (Incardona *et al.*, 2009 ▸) and *autoPROC* (Vonrhein *et al.*, 2011 ▸) processing files generated by the automatic pipeline at BioMAX were used. The data were then scaled and merged using *AIMLESS* (Evans & Murshudov, 2013 ▸) within the *CCP*4 program suite (Agirre *et al.*, 2023 ▸). For the UrdA′P-RT sulfate complex the data were scaled and merged using the *autoPROC* software (Vonrhein *et al.*, 2011 ▸) within the BioMAX pipeline and the resulting data file was inspected and used for further structure determination. *Phaser* (McCoy *et al.*, 2007 ▸) and *AutoBuild* (Terwilliger *et al.*, 2008 ▸) within *Phenix* (Liebschner *et al.*, 2019 ▸) were used for molecular replacement (with PDB entry 6t87 as a search model) and model building. Further refinement and model building was performed in *phenix.refine* (Afonine *et al.*, 2012 ▸) and *Coot* (Emsley *et al.*, 2010 ▸). The structures of UrdA′S-RT and UrdA′P-cryo with sulfate bound were refined using anisotropic *B* factors for all atoms. All other structures were refined using isotropic *B* factors for all atoms. Riding hydrogens were added in the refinement of the UrdA′P-cryo and UrdA′S-RT structures with sulfate bound. All of the amino-acid residues could be modeled, except for the C-terminal 6×His tag. Also, an FAD cofactor (in an oxidized state) and substrate/product were modeled based on unambiguous electron densities. During data analysis and refinement, we focused on the active site, specifically Arg411 and the anion-binding site. Structures were refined and built with alternative conformations for Arg411, and these were coupled with nearby water molecules through occupancies before deciding on the final model. For these calculations we used omit polder maps (Liebschner *et al.*, 2017 ▸) to provide an unbiased electron density for Arg411, the focus of this study. The data and refinement statistics are provided in Table 1 ▸. Structure figures were prepared in *PyMOL* (Schrödinger). The movement of the clamp domain between the structures was calculated using the *DynDom* online software (Veevers & Hayward, 2019 ▸).

### 2.3. Differential scanning fluorimetry

Differential scanning fluorimetry (nanoDSF) was performed using a Prometheus NT.48 (NanoTemper), where each sample was loaded into a glass capillary. The purified wild-type UrdA′ and R411A variant (Venskutonytė *et al.*, 2021 ▸) were used at a final concentration of 1 mg ml^−1^ with and without substrate and product at 1 m*M* final concentration and with and without sodium phosphate (10 and 100 m*M*) or sodium sulfate (100 m*M*). The samples were prepared in a buffer consisting of 20 m*M* HEPES pH 7.0, 150 m*M* NaCl. Each sample was prepared in triplicate and the standard error of the mean (SEM) was calculated for each triplicate. The data were analyzed using the *PR.ThermControl* v.2.0.4 software. The pre-processed data generated by the software were used to plot the ratio of 350/330 nm and first derivative versus temperature in Python. A few missing values were filled by linear interpolation using the interpolate method in the pandas library for Python. The average *T*_m_ values were plotted (Fig. 4) and are listed in Table 2 ▸.

## 3. Results

### 3.1. Room-temperature X-ray structure of the UrdA′–substrate complex

**Figure 1:**
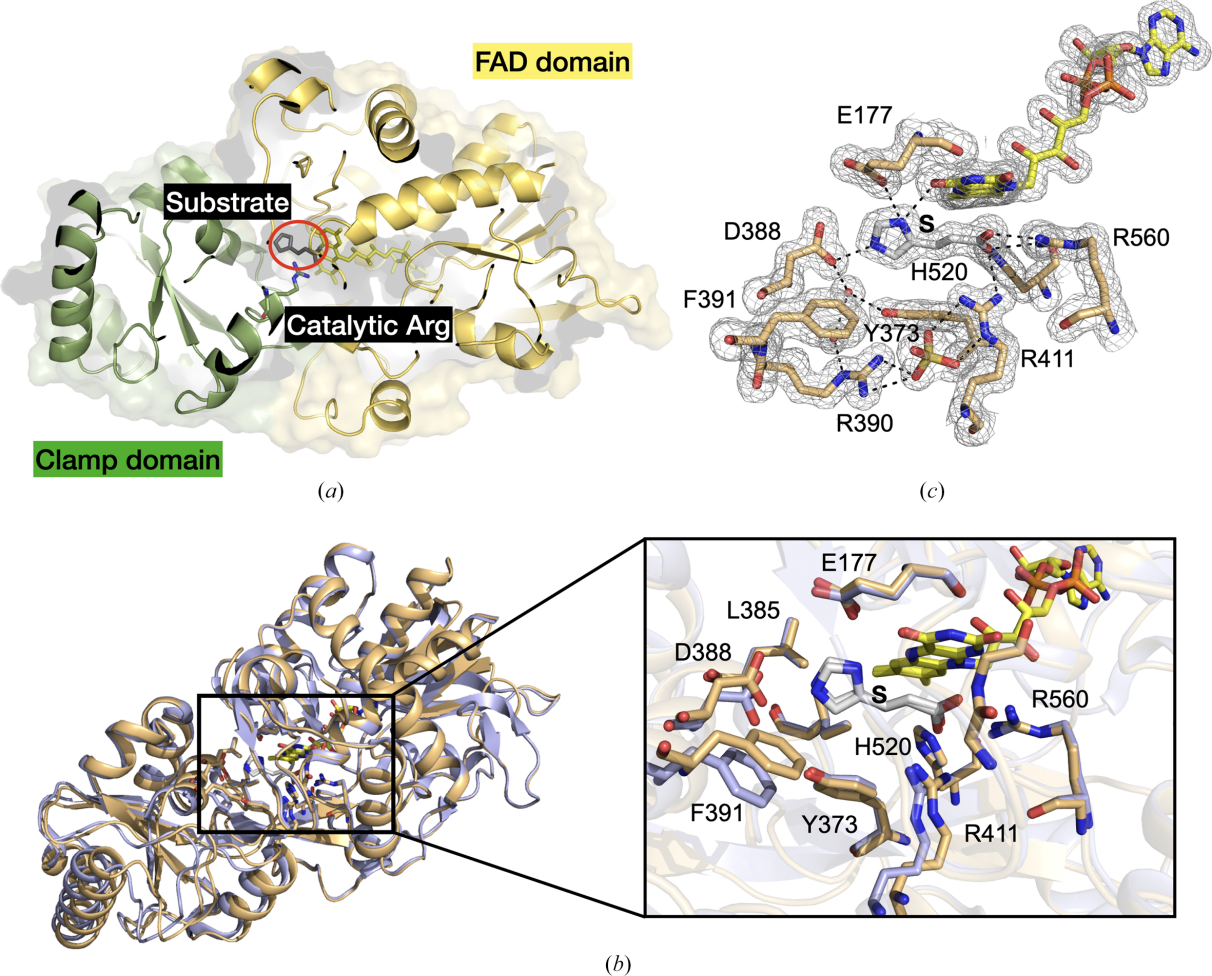
Structure of UrdA at cryogenic and room temperatures. (*a*) Cartoon representation of the RT structure of UrdA showing the FAD domain (yellow) and clamp domain (green), with bound substrate (red circle) and the catalytic Arg411 shown as sticks. (*b*) Superimposition of the UrdA′S-RT (beige) and UrdA′S-cryo (light purple; PDB entry 6t87) structures using the FAD domain. An enlargement of the active site is also shown. Substrate is marked S. (*c*) Active site of the UrdA′S-RT structure with a 2*mF*_o_ − *DF*_c_ map shown at 1σ and hydrogen bonds shown as dashed lines. Substrate (urocanate) is marked S.

To complement our previously obtained X-ray data on UrdA′ under cryogenic conditions (Venskutonytė *et al.*, 2021 ▸) we analyzed UrdA′ using RT X-ray crystallography. UrdA′ crystals were reproduced (Venskutonytė *et al.*, 2021 ▸) and demonstrated sufficient stability for room-temperature data collection. First, we solved a structure in complex with the substrate urocanate (UrdA′S-RT) in the presence of ammonium sulfate. The unit-cell dimensions were slightly larger for this structure compared with the UrdA′ structure with substrate under cryo-conditions (UrdA′S-cryo), with the *a* and *b* axes being 126.3 Å for the RT crystals and 123.3 Å for the cryocooled crystals, respectively (PDB entry 6t87). When comparing the overall structures, they are highly similar and the C^α^ atoms align with an r.m.s.d. of 0.4 Å (*PyMOL*). UrdA′ consists of a stable FAD domain and a flexible clamp domain (Fig. 1 ▸*a*), which undergoes a large movement upon the binding of substrate (Venskutonytė *et al.*, 2021 ▸). When overlaying the FAD domains of the UrdA′S-RT and UrdA′S-cryo structures, a small difference in the mobile clamp domain conformation can be seen (Fig. 1 ▸*b*), *i.e.* the clamp domain is more closed by 5° (as calculated by *DynDom*; Veevers & Hayward, 2019 ▸). Overall, the substrate-binding site is similar (Fig. 1 ▸*b*) and is well defined (Fig. 1 ▸*c*), but due to the domain movement some residues, such as Phe391, move closer to the substrate. Interestingly, this aligns more with the cryocooled structure of UrdA′ in complex with the product (UrdA′P-cryo; Supplementary Fig. S1).

### 3.2. Room-temperature X-ray structure of the UrdA′–product complex

**Figure 2:**
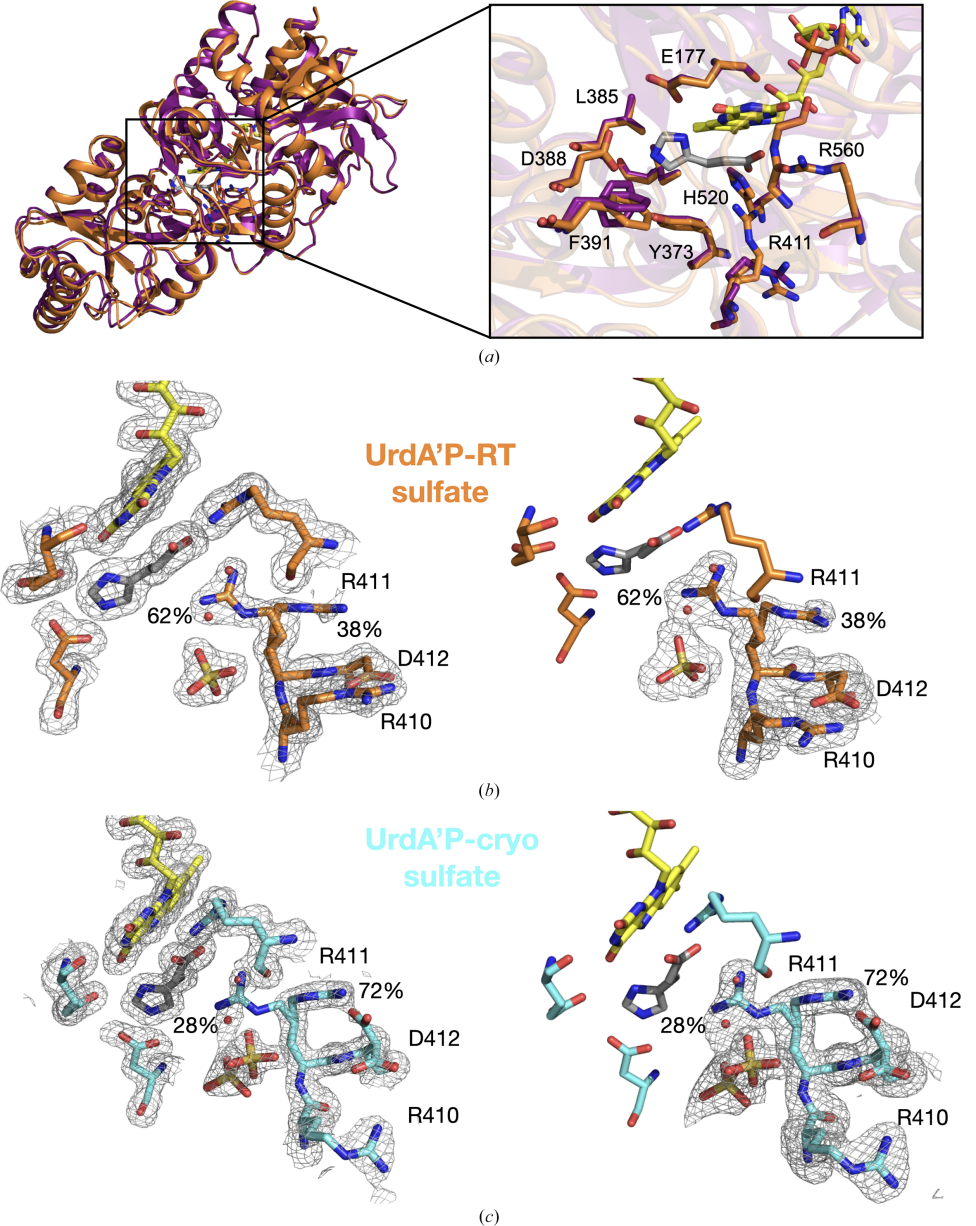
UrdA′P structures at cryogenic and room temperatures. (*a*) Cartoon representation of the superposition of the UrdA′P-RT (orange) and UrdA′P-cryo (purple; PDB entry 6t88) structures using the FAD domain and an enlargement of the active site. (*b*) Left: 2*mF*_o_ − *DF*_c_ map contoured at 1σ. Right: *mF*_o_ − *DF*_c_ omit polder map for residues 410–412 and the sulfate and water molecules contoured at 3σ for the UrdA′P-RT structure with bound sulfate. (*c*) Left: 2*mF*_o_ − *DF*_c_ map contoured at 1σ. Right: *mF*_o_ − *DF*_c_ omit polder map shown for residues 410–412 and the sulfate and water molecules contoured at 3σ for the UrdA′P-cryo structure. Occupancies for each of the Arg411 alternatives are indicated by the percentage values given.

To gain a comprehensive understanding of how RT data collection influences structure, we solved a RT crystal structure of UrdA′ in complex with the product imidazole propionate (UrdA′P-RT) crystallized in a condition containing ammonium sulfate. As observed for the UrdA′S-RT crystals, the UrdA′P-RT crystals also had longer unit-cell dimensions (the *a* and *b* axes increased by ∼2%) compared with a previously published cryo structure (UrdA′P-cryo). The RT structure is virtually identical to the cryo structure and aligns with an r.m.s.d. of 0.3 Å for all C^α^ atoms. Upon superimposing the FAD domains, the clamp domains are well aligned and *DynDom* analysis shows only a 2° domain rotation between the two structures, with UrdA′P-RT being in a more closed conformation (Fig. 2 ▸*a*). Interestingly, when overlaying UrdA′P-RT with UrdA′S-RT they align exactly, with both being more similar to the previously published UrdA′P-cryo structure (PDB entry 6t88) than to UrdA′S-cryo (PDB entry 6t87; Supplementary Fig. S1). Overall, the binding-site residues are also in very similar positions when comparing the UrdA′P-RT and UrdA′P-cryo structures, except for Phe391, which we have previously suggested to be part of the hydrophobic lid that is involved in product release and adopts a single conformation, while in a previous high-resolution cryocooled structure it is very flexibile (Fig. 2 ▸*a*).

This is interesting as usually the opposite is observed: RT structures reveal higher rotameric variation than structures obtained by cryocooling the crystals. This may be due to cryocooling effects, as described in Fischer *et al.* (2015 ▸), or in this particular case due to a glycerol molecule bound in the vicinity of Phe391 upon applying cryoprotectant prior to crystal cooling. Another major change in the UrdA′P-RT structure compared with the UrdA′P-cryo structure is the conformation of Arg411. We have previously shown that this catalytic residue adopts two distinct conformations, hereafter called ‘conformation S’ and ‘conformation P’, when bound to substrate or product, respectively, and demonstrated that substituting this residue by alanine completely abolished the activity of the enzyme (Venskutonytė *et al.*, 2021 ▸). Surprisingly, in the UrdA′P-RT structure Arg411 largely adopts conformation S. However, there is additional electron density for conformation P, and refinement of the two rotamers results in occupancies of 62% for the S and 38% for the P conformation (Fig. 2 ▸*b*). We have also calculated omit polder maps, which further show that the alternative rotamers are present (Fig. 2 ▸*b*). Thus, it seems that in UrdA′P-RT Arg411 is allowed more flexibility, while in the cryo structures the equilibrium is pushed towards one conformation. This could, however, also be an effect of other differences in the vicinity of Arg411. Indeed, there are changes in the adjacent residues Arg410 and Asp412; these residues showed high flexibility in the UrdA′P-cryo structure (PDB entry 6t88) and indicated the presence of multiple rotamers. However, in the UrdA′P-RT structure both Arg410 and Asp412 clearly show one distinct conformation and electron densities are well defined (Fig. 2 ▸*b*). In fact, in the UrdA′P-RT structure Arg410 and Asp412 are facing each other and form a hydrogen bond, while in the UrdA′P-cryo structure these residues are located away from each other and one of the Asp412 rotamers instead binds to Arg411 (Supplementary Fig. S2). It is important to note that Arg410 is positioned near the symmetry axis at the dimer interface with the neighboring molecule. This might be a critical area when it comes to crystal cryo effects and, as mentioned previously, the cooling shrinks the unit cell, meaning that the packing of the molecules within the lattice will be affected.

### 3.3. Sulfate ion in the binding site affects the conformation of Arg411

Commonly, the crystallization conditions for UrdA′ contain ammonium sulfate, and a sulfate ion was found next to the binding site of Arg411 in the previously published UrdA′S-cryo structure and in both UrdA′S-RT and UrdA′P-RT presented here (Figs. 1 ▸ and 2 ▸). The previously published UrdA′P-cryo structure was, however, crystallized in the presence of ammonium chloride, and in fact a chloride ion and a glycerol molecule were found in the active site in the region that becomes available when Arg411 adopts the P conformation, which is in contrast to the UrdA′P-RT structure where Arg411 adopts the S conformation. This raises the possibility that the sulfate ions may influence the positioning of Arg411, potentially acting as stabilizing agents. To further analyze this and to allow direct comparison between the RT versus cryo product complexes, we determined the UrdA′P-cryo structure from a crystal grown in the presence of sulfate. In this structure, Arg411 still largely adopts the P conformation previously seen in the cryo structure. However, there is electron density to support the presence of the substrate-facing rotamer in agreement with the S conformation, and refinement of both rotamers resulted in 28% occupancy for the S-like conformation. In addition, when inspecting the omit polder maps, the densities indicate two distinct conformations (Fig. 2 ▸*c*). The presence, even though at low occupancy, of the S-like conformation is further supported by two apparent alternatives for Asp412, as one of these less pronounced conformations would clash with Arg411 when it is in the P conformation. This result further supports cryo-conditions favoring the adoption of a P conformation by Arg411; however, it indicates that the presence of sulfate ion in the active site plays a role in the positioning of Arg411 and might stabilize it in the S conformation, even with the product bound, and these interactions are much more pronounced in the RT structures.

### 3.4. Room-temperature X-ray structures of UrdA′ crystallized without sulfate

**Figure 3:**
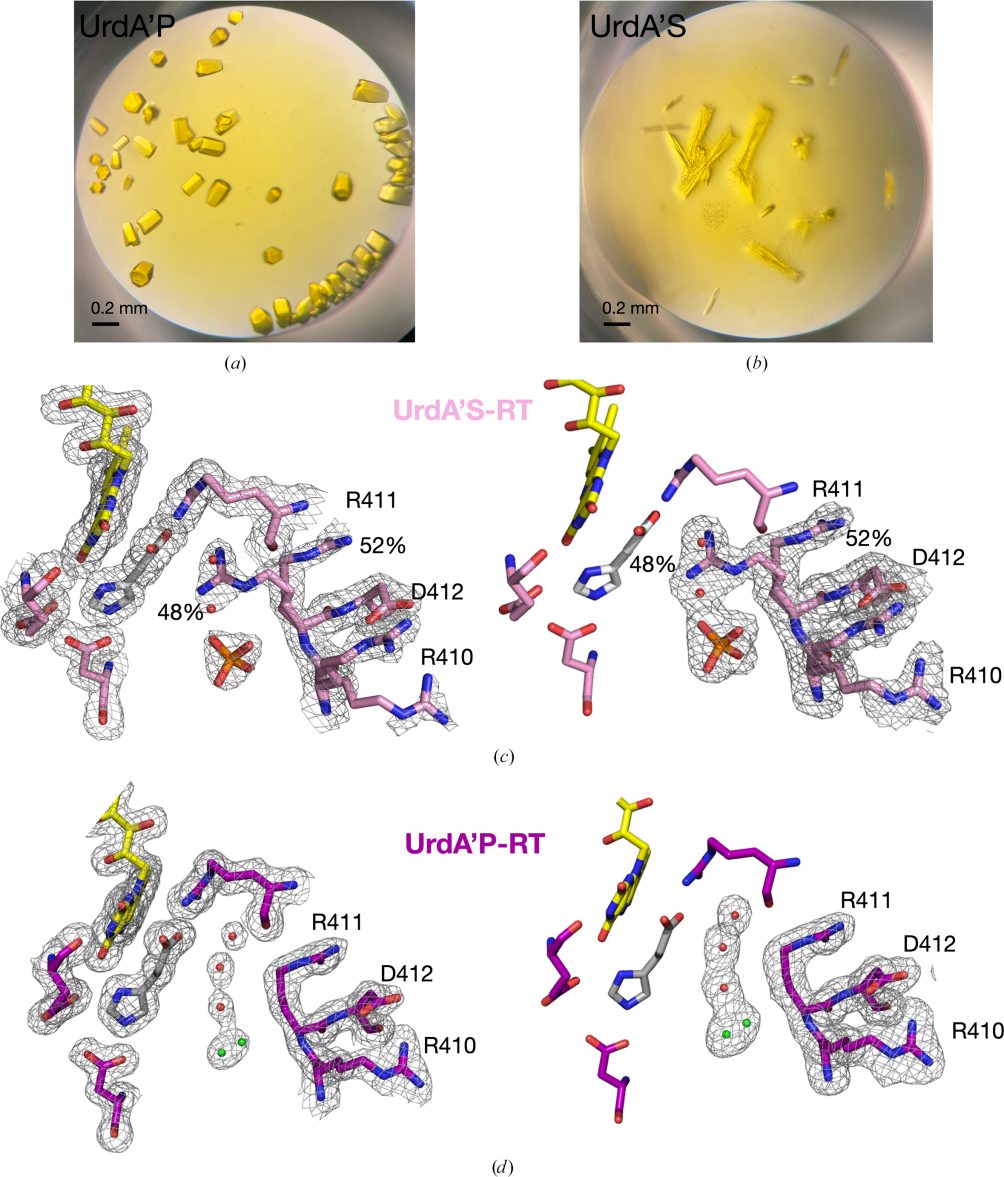
UrdA room-temperature X-ray structures obtained in the presence of citrate. (*a*) UrdA′P crystals (2 µl drop) grown in ammonium citrate. (*b*) UrdA′S crystals (2 µl drop) grown in ammonium citrate. (*c*) Left: 2*mF*_o_ − *DF*_c_ map shown at 1σ. Right: *mF*_o_ − *DF*_c_ omit polder map shown for residues 410–412 as well as the phosphate and water molecules contoured at 3σ for the UrdA′S-RT structure under citrate conditions. Occupancies for each of the Arg411 alternatives are indicated by percentage values. (*d*) Left: 2*mF*_o_ − *DF*_c_ map shown at 1σ. Right: *mF*_o_ − *DF*_c_ omit polder map shown for residues 410–412 as well as for the chloride ion and water molecules contoured at 3σ for the UrdA′P-RT structure under citrate conditions.

To gain deeper insight into the influence of sulfate ions on the active-site environment and their effect on the conformation of Arg411, we crystallized UrdA′ in complex with substrate or product under crystallization conditions devoid of sulfate ions. Besides PEG, UrdA′ also requires an ammonium salt precipitant for crystallization; thus, ammonium citrate was used as citrate ions would be too large to bind in the equivalent site as seen for sulfate. The crystals with product appeared earliest after two days and were of a similar size and quality as expected based on previous experience (Fig. 3 ▸*a*).

Crystals with urocanate (substrate) took longer to grow, were thinner and more clustered (Fig. 3 ▸*b*). However, both complexes diffracted well at RT; full datasets were collected and structures were determined at 1.8 and 1.7 Å resolution for the substrate and product complexes, respectively. The active site of UrdA′S-RT shows both S and P conformations for Arg411 in 2*mF*_o_ − *DF*_c_ and omit polder maps (Fig. 3 ▸*c*) and, surprisingly, in the sulfate binding site we observed a density consistent with a sulfate ion or a similar species. As sulfate ions were not added to the crystallization buffer or to previous purification steps, we modeled a phosphate ion (Fig. 3 ▸*c*), as sodium phosphate buffer was used in the first steps of purification of UrdA′. This result would imply that the substrate complex requires a cofactor, such as sulfate or phosphate, to create an interaction network via Arg411 and to stabilize Arg411 in the S conformation. This is further supported by the occupancy refinement of the two alternative Arg411 conformations and the phosphate molecule. The refinement indicates that phosphate is partially present (it refines with an occupancy of 68%), while Arg411 is almost equally divided between the S and P conformations (Fig. 3 ▸*c*). Notably, in our experience it is difficult to produce crystals of UrdA′S without sulfate, which is in contrast to UrdA′P. For instance, we can routinely grow good-quality UrdA′P crystals in the presence of ammonium chloride, but this cannot be achieved for UrdA′S. This might be due to chloride binding in the anion site and thereby displacing the phosphate, which is likely to be necessary for UrdA′S complex stabilization. Moreover, we have collected an X-ray data set for UrdA′S-cryo grown with citrate. The unit-cell dimensions *a* and *b* decreased to 124.6 Å, compared with 126.2 Å for the RT citrate structure, and electron density most likely representing a phosphate ion is present in the substrate-binding site. Interestingly, compared with UrdA′S-RT we observe a shift in occupancy distribution, with the P conformation of Arg411 becoming the dominant conformation (72%; Supplementary Fig. S3). This further indicates that a stabilizing modulator such as phosphate is needed for Arg411 to adopt the S conformation. However, the fact that the P conformation is more prominent under cryogenic conditions while it is more equally distributed between the two conformations at RT under the same condition points to the suggestion that the P conformation represents a low- energy conformation which is favored upon cooling. In the UrdA′P-RT structure with citrate, the electron density does not support the presence of the S conformation, and Arg411 clearly only adopts the P conformation (Fig. 3 ▸*d*). Further, we do not see a clear density that could be assigned to phosphate in the binding site, and we modeled a chloride ion in this location instead. In both cases, UrdA′S and UrdA′P, the modeled ions, phosphate and chloride, respectively, participate in extensive interactions with surrounding residues and water molecules (Supplementary Fig. S4)

### 3.5. Phosphate and sulfate ions contribute to UrdA′ stability via interactions with Arg411

To investigate whether phosphate or sulfate ions do indeed stabilize UrdA′ via the Arg411 residue, we performed a thermal stability assay using differential scanning fluorimetry (nanoDSF). To differentiate whether the anion effect is Arg411-dependent, we included an R411A UrdA′ mutant in the assays. UrdA′_WT_ and UrdA′_R411A_ were assayed in the presence of substrate or product and supplemented with either sodium phosphate or sodium sulfate and the melting temperatures (*T*_m_) were then measured (Table 2 ▸).

**Figure 4:**
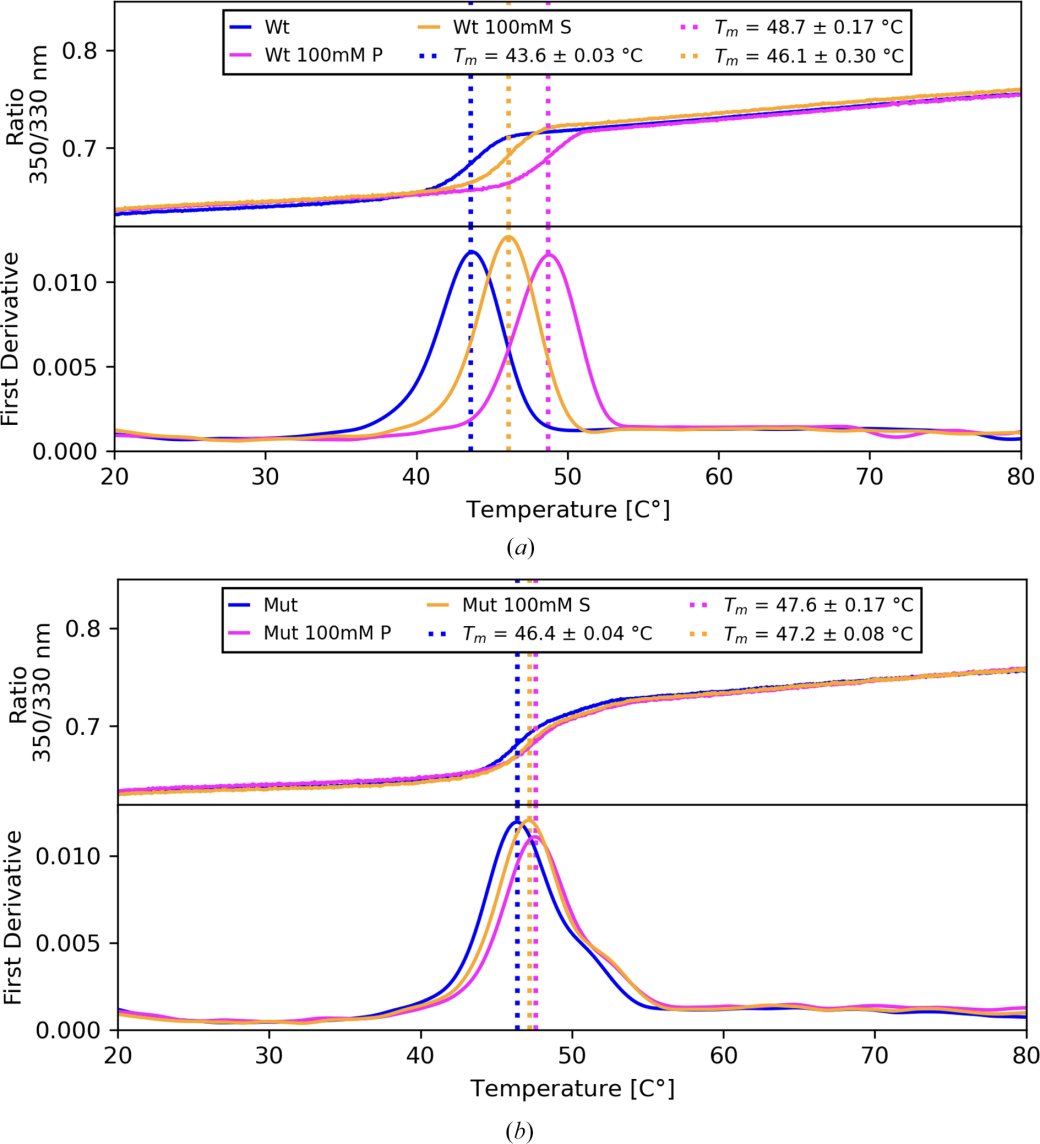
Thermal stability nanoDSF measurements for (*a*) UrdA′_WT_ (Wt) and (*b*) UrdA′_R411A_ (Mut) in the presence of sulfate and phosphate. P denotes phosphate and S denotes sulfate.

Interestingly, without anions UrdA′_R411A_ is more stable than UrdA′_WT_ by 3°C, but after the addition of anions we observed a clear stabilizing effect on UrdA′_WT_, with phosphate increasing the *T*_m_ by up to 6°C, while sulfate had a milder effect, with a 3°C increase in *T*_m_ at 100 m*M* (Fig. 4 ▸*a*). To exclude the general effect of ionic strength we included a control with 300 m*M* NaCl, but this did not result in a change in *T*_m_. On the other hand, the anion effect on UrdA′_R411A_ was much less pronounced, with only a 1°C increase in *T*_m_ (Fig. 4 ▸*b*).

These results suggest that protein stabilization is achieved by an anionic interaction with Arg411 and that phosphate ions have a more pronounced effect than sulfate. From the crystal structures we know that the anion binding site is formed in UrdA′S and that phosphate/sulfate form interactions with Arg411 and Arg390. However, since the substrate-free UrdA′ also showed an increase in *T*_m_ upon the addition of anions, it appears that the anion can stabilize UrdA′ via Arg411 without ligand binding. Indeed, in the apo structure (PDB entry 6t86; Venskutonytė *et al.*, 2021 ▸) a sulfate ion is found in the active site forming a salt bridge to Arg560 and a hydrogen bond to His520, residues which otherwise coordinate to the substrate or product. This sulfate ion further forms a contact to Arg411, basically bridging the clamp and FAD domains together. Interestingly, there is another sulfate ion between Arg411 and Arg410 in the apo structure, interacting with both residues. Overall, these results show that phosphate and sulfate ions can stabilize UrdA′ via interactions involving the catalytic Arg411.

## 4. Discussion

**Figure 5:**
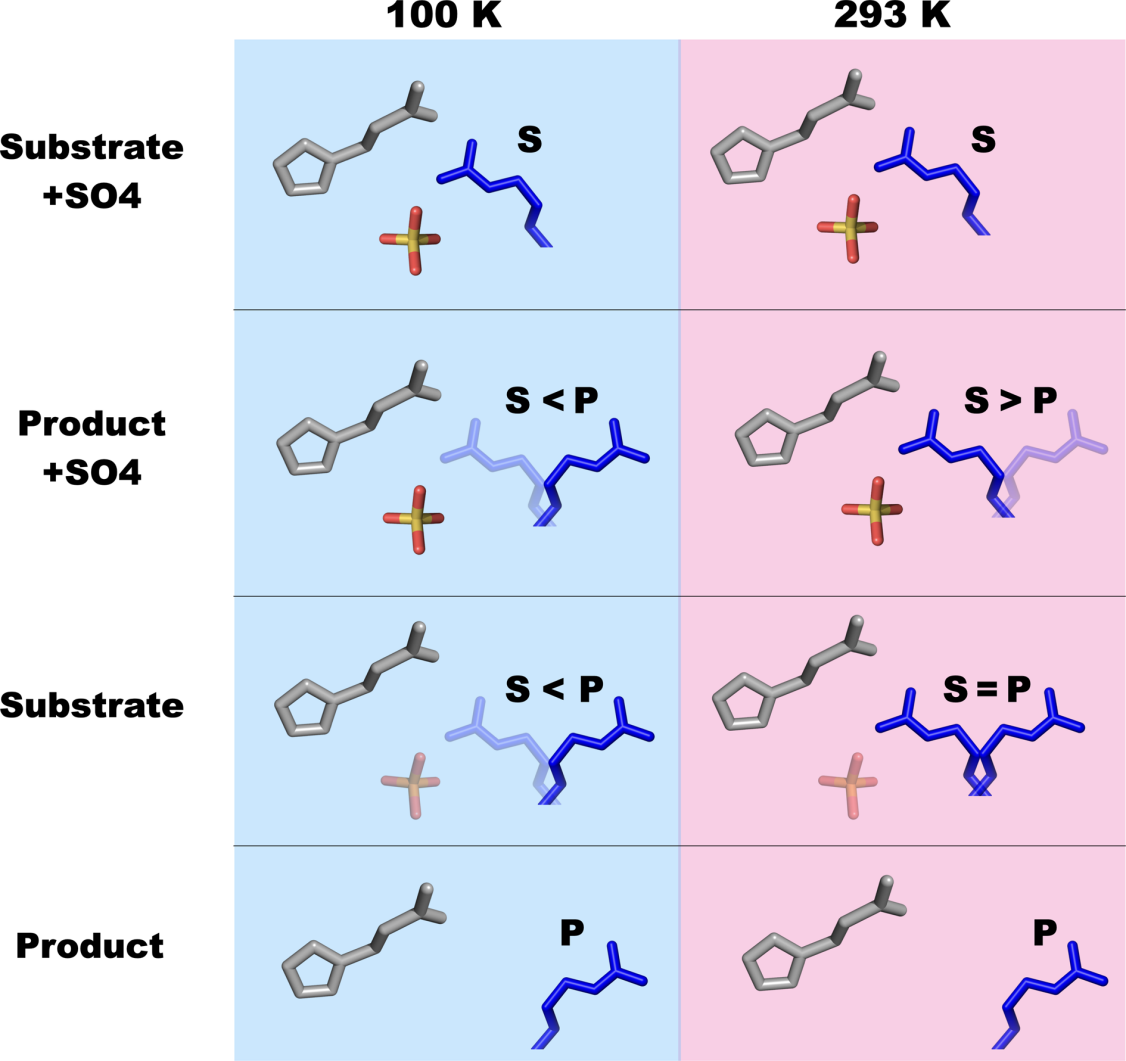
Schematic summary of the Arg411 occupancy distribution in the presence and absence of sulfate ions for the substrate and product complex under cryogenic versus RT conditions.

We have previously shown that the major catalytic residue Arg411 in UrdA switches between the substrate (urocanate)-bound state, with Arg411 facing towards the substrate, and the product-like state (imidazole propionate), undergoing a large conformational change in the process. Substituting this residue with alanine completely abolishes the activity of the enzyme (Venskutonytė *et al.*, 2021 ▸). To investigate potential structural heterogeneity across the catalytic cycle, we have collected RT X-ray data on UrdA, revealing that unlike in the cryo structures Arg411 is mostly in the substrate-like conformation (S conformation) in the presence of both product and substrate. We also observe a sulfate ion in the active site interacting with Arg411 when Arg411 is in the S conformation. We postulate that the UrdA′P-RT structure obtained in the presence of sulfate reveals biasing of the Arg411 conformation by the sulfate ion towards the substrate conformation. However, in the UrdA′P structure under cryogenic conditions the influence of the sulfate ions was markedly reduced and Arg411 largely adopts the expected P conformation, with an occupancy of ∼70% compared with 40% as seen in the RT structure (Fig. 5 ▸). Possible reasons could be that cryocooling reduces the anion-binding affinity by affecting the binding geometry, which in turn reduces the sulfate biasing in a similar manner as reported for changes in ligand binding upon cryocooling (Fischer *et al.*, 2015 ▸). Another possibility might be that cryocooling traps an energy-minimized state, as the P conformation is preferred for UrdA′P-cryo. This argues for the importance of collecting X-ray data at both room and cryo temperatures in order not to miss important dynamics of the system studied.

To further confirm the role of sulfate ions in affecting the Arg411 conformation, we have solved UrdA′S-RT and UrdA′P-RT structures using crystals obtained in the absence of sulfate. Interestingly, in the UrdA′S-RT structure we still observed density in the same position agreeing with a sulfate or phosphate molecule at partial occupancy and modeled a phosphate based on the composition of the protein purification buffer. In the UrdA′P-RT structure the density in the binding site was not in the shape of a tetrahedral anion and chloride ion appeared to be a better fit. Most importantly, Arg411 adopts two conformations at equal occupancies (48% S conformation and 52% P conformation) in the UrdA′S-RT structure, while Arg411 in UrdA′P-RT is 100% in the P conformation (Fig. 5 ▸). However, cryocooling the UrdA′S-cryo (without sulfate) crystal altered the Arg411 occupancy distribution in favor of the P conformation, further illustrating that this conformation also becomes predominant under cryogenic conditions in UrdA′S-cryo, without anion supplementation. Interestingly, it is much easier to achieve good-quality crystals for UrdA′P using ammonium citrate or ammonium chloride, while crystals do not readily appear for UrdA′S in conditions lacking sulfate ions, which would argue in favor of the role of the anion in stabilizing Arg411 in the substrate complex. The neighboring residue to the catalytic Arg411 is also an arginine: Arg410, which lies in the interface of the crystallographic dimer facing towards the equivalent residue in the symmetry-related molecule. Arg410 often shows flexibility in the structures (for example multiple rotamers) as Arg411 affects the rotamer of Asp412, which can either interact with Arg410 or point away, and this in turn can affect the rotamer conformation of Arg410. These movements could subsequently affect lattice formation and crystallization. Alternatively, without the stabilizing anion interactions Arg411 samples the two conformations, which might not be energetically favorable in UrdA′S and possibly impair crystallization by preventing the local ordering required for nucleation. Collectively, our findings confirm that the conformations previously described as substrate-like and product-like correspond to biologically meaningful rotameric states and suggest that an oxyanion plays a critical role in maintaining a stable substrate complex. We also show that the Arg411 dynamics can be influenced by crystal cryocooling, favoring the occupancy shift towards the P conformation (Fig. 5 ▸).

This raises the question of the mechanistic role of the anion-binding site. Interestingly, in the related enzyme fumarate reductase there is a glutamate residue participating in the proton-transfer pathway that overlaps exactly with the sulfate in the UrdA structures and interacts with an equivalent catalytic arginine (Pankhurst *et al.*, 2006 ▸). This glutamate residue is not present in UrdA, so the question is whether a phosphate could be part of the proton-transfer pathway instead. To our knowledge there are no reported examples of this in the family of oxidoreductases. Possibly, the observed anion in UrdA mimics the role of a catalytic glutamate present in related fumarate reductases *in vitro*, while *in vivo* this role may be achieved by other means, different modulating molecules or simply a water network. Phosphodianion binding energy is on the other hand often utilized for active-site formation while being part of the substrate in glycerol-3-phosphate dehydrogenase, triosephosphate isomerase and other central metabolic enzymes (Fernandez *et al.*, 2021 ▸; Mydy *et al.*, 2019 ▸; Zhai *et al.*, 2015 ▸). In fact, it was shown that exogeneous phosphite dianion increases the turnover by 700-fold in triosephosphate isomerase (Amyes & Richard, 2007 ▸). Interestingly, it has also been suggested that the cofactor pyridoxal 5′-phosphate plays a role via its phosphodianion in enzymatic catalysis of carbon deprotonation (Richard *et al.*, 2011 ▸). When it comes to free phosphate ion acting as a stabilizing agent within the binding site, this has been seen in other enzymes, such as the plant nuclease TBN1 (Koval’ *et al.*, 2013 ▸; Stránský *et al.*, 2015 ▸). Also, in purine nucleoside phosphorylase (PNP) the phosphate dianion was shown to bind in the active site and to play a role in the catalytic triad, acting as a nucleophile (Erion *et al.*, 1997 ▸) and, similar to our observations in UrdA′, interacting with arginine residues in the binding site (Chaikuad & Brady, 2009 ▸). The phosphate binding site in PNPs is highly conserved and can also be occupied by sulfate in crystal structures (de Azevedo *et al.*, 2003 ▸; Pugmire & Ealick, 2002 ▸). Taken together, it seems that phosphate/sulfate ions can have diverse roles throughout different enzyme classes, including purely structural and/or chemically active. While in UrdA structures the phosphate/sulfate ion is not in the vicinity of residues that could deprotonate the phosphate as in the PNPs, it is possible that the phosphate site was evolutionarily retained and contributes to catalysis by orienting the catalytic arginine.

Moreover, the anion-binding site could also indicate that UrdA binds or maybe even catalyses other substrates containing a phosphate moiety. While UrdA has been characterized as a bona fide urocanate reductase, it is possible that it could reduce other metabolites or just have a binding affinity. The Rossmann-fold motif is highly preserved in evolution and is present in a wide range of metabolic enzymes that can bind a variety of ligands (Medvedev *et al.*, 2021 ▸). Overall, enzyme promiscuity had been observed for different enzymes and is an evolutionary mechanism (Fuentes-Ugarte *et al.*, 2025 ▸). While promiscuity can occur by different substrates sharing the same active site, it can also be dependent on other factors, such as protonation states of catalytic residues, water-network variations, alternative cofactors or separate subsites within the active site (Khersonsky & Tawfik, 2010 ▸). The latter can be illustrated by the interesting case of serum paraoxanase: besides the native lactone hydrolysis activity, it is known to also have a phosphotriesterase activity due to different residues in the subsite (Khersonsky & Tawfik, 2010 ▸). Among flavoenzymes, which are very versatile in general (Walsh & Wencewicz, 2013 ▸), nitroreductase-like enzymes were shown to be able to reduce nitro groups in many different compounds as well as to catalyse C=C reduction (Luján *et al.*, 2024 ▸). UrdA is one of many enzymes participating in the histidine metabolic pathway. Some of the metabolites contain phosphate moieties, such as imidazole-3-glycerol phosphate and l-histidinol phosphate. Similar metabolites could in fact occupy the active site of UrdA′, exploring both the imidazole-binding residues and the anion-binding site. While it is not clear whether such ligands could serve as additional substrates, this would be interesting to investigate in a future study. Moreover, the presence of the anion site could serve as a starting point for inhibitor design targeting UrdA′. The space occupied by the substrate and anion-binding site could be explored in structure-based drug design. Lastly, we should also bear in mind that to date all structural studies were performed on UrdA′ as a two-domain construct, but in the full-length enzyme the N-terminal domain might contribute to also stabilizing the active site and reveal other structural details.

In conclusion, our studies of UrdA′ using RT crystallo­graphy provide further insight into the complexity of active-site dynamics and show that Arg411 samples the substrate-like and product-like conformations, where an active-site anion enforces the substrate-like conformation rotamer. Comparison with the cryogenic structures reveals that the biologically relevant conformations might be favored by cryogenic conditions, possibly due to capturing the residue in the local energy minima, the end-point state. These studies also indicate that UrdA has a biologically relevant anion, likely phosphate, binding site within its active pocket. Overall, our study suggests that when possible both cryogenic and RT X-ray data should be collected for an improved biological interpretation of structural results.

## Supplementary Material

PDB reference: UrdA′S-RT, sulfate-bound, 9tk1

PDB reference: UrdA′P-RT, sulfate-bound, 9tk3

PDB reference: UrdA′S, 9tk5

PDB reference: UrdA′P, 9tk6

PDB reference: UrdA′P-cryo, sulfate-bound, 9tk7

PDB reference: UrdA′S-cryo, 9tk8

Supplementary Figures. DOI: 10.1107/S2059798326003360/cb5158sup1.pdf

## Figures and Tables

**Table 1:** Crystallographic data and refinement statistics

Dataset	UrdA′S-RT (sulfate)	UrdA′P-RT (sulfate)	UrdA′P-cryo (sulfate)	UrdA′S	UrdA′P	UrdA′S-cryo
PDB code	9tk1	9tk3	9tk7	9tk5	9tk6	9tk8
Data collection						
Wavelength (Å)	0.9763	0.9763	0.9794	0.9763	0.9763	0.9763
Resolution (Å)	109.37–1.60 (1.63–1.60)	63.133–1.93 (1.96–1.93)	66.28–1.50 (1.53–1.50)	45.84–1.80 (1.864–1.80)	45.87–1.70 (1.761–1.70)	45.43–1.90 (1.94–1.90)
Space group	*P*3_1_21	*P*3_1_21	*P*3_1_21	*P*3_1_21	*P*3_1_21	*P*3_1_21
*a*, *b*, *c* (Å)	126.293, 126.293, 66.868	126.265, 126.265, 66.782	124.143, 124.143, 66.279	126.176, 126.176, 66.729	126.239, 126.239, 66.782	124.55, 124.55, 66.41
α, β, γ (°)	90, 90, 120	90, 90, 120	90, 90, 120	90, 90, 120	90, 90, 120	90, 90, 120
Total reflections	833616 (40398)	250486 (11958)	1884032 (93343)	577129 (34394)	690034 (36250)	362570 (23882)
Unique reflections	80972 (3946)	46321 (2289)	93985 (4591)	56836 (3321)	67479 (3561)	46942 (3008)
Multiplicity	10.3 (10.2)	5.4 (5.2)	20.0 (20.3)	10.2 (10.4)	10.2 (10.2)	7.7 (7.9)
Completeness (%)	100 (100)	99.8 (100)	100.0 (100.0)	100.0 (100.0)	100.0 (100.0)	99.99 (99.99)
Mean *I*/σ(*I*)	15.3 (1.4)	9.0 (2.5)	21.9 (2.4)	10.2 (1.7)	12.7 (2.2)	11.2 (1.7)
*R*_merge_	0.078 (1.799)	0.202 (1.956)	0.081 (1.719)	0.120 (1.116)	0.102 (1.036)	0.097 (1.513)
CC_1/2_	0.999 (0.561)	0.988 (0.321)	1.000 (0.772)	0.998 (0.826)	0.999 (0.798)	0.998 (0.760)
Refinement						
Resolution (Å)	32.01–1.60	31.99–1.93	53.76–1.50	31.97–1.80	31.56–1.70	31.62–1.90
Reflections	80928 (2735)	46126 (2749)	93934 (3078)	56721 (2777)	67434 (2766)	46840 (2720)
*R*_work_/*R*_free_	0.1087/0.1392	0.1464/0.1763	0.1129/0.1392	0.1491/0.1690	0.1426/0.1613	0.1562/0.1981
No. of atoms						
Protein	3554	3508	3516	3513	3507	3463
Ligands	69	69	121	69	66	69
Solvent	293	330	535	280	314	417
R.m.s. deviations						
Bond lengths (Å)	0.008	0.007	0.009	0.006	0.006	0.006
Bond angles (°)	01.00	0.79	1.05	0.80	0.80	0.75
Ramachandran plot (%)						
Favored	97.79	97.57	97.57	97.57	98.01	97.35
Allowed	2.21	2.43	2.43	2.43	1.99	2.65
Outliers	0.00	0.00	0.00	0.00	0.00	0.00
Rotamer outliers (%)	0.53	0.27	0.27	0.54	0.54	0.56
Clashscore	1.81	1.41	0.55	1.26	1.12	1.57
*B* factors (Å^2^)						
Protein	26.78	20.53	21.49	26.31	23.96	37.77
Ligands	18.35	12.47	27.25	18.35	15.13	31.84
Solvent	41.49	31.68	37.26	37.36	36.37	44.87

**Table 2:** *T*_m_ values measured by nanoDSF in triplicate (unless stated otherwise) for UrdA′_WT_ and UrdA′_R411A_ in the presence and absence of substrate/product and anions

Protein	Ligand	Anion	*T*_m_ ± SEM (°C)
UrdA′_WT_	—	None	43.6 ± 0.03 (*n* = 4)
		10 m*M* phosphate	46.7 ± 0.18
		100 m*M* phosphate	48.7 ± 0.17
		100 m*M* sulfate	46.1 ± 0.30
	Urocanate	None	43.6 ± 0.11 (*n* = 4)
		10 m*M* phosphate	46.8 ± 0.08
		100 m*M* phosphate	49.0 ± 0.12
		100 m*M* sulfate	45.7 ± 0.13
	Imidazole propionate	None	43.6 ± 0.02 (*n* = 4)
		10 m*M* phosphate	46.8 ± 0.05
		100 m*M* phosphate	49.1 ± 0.18
		100 m*M* sulfate	45.8 ± 0.03 (*n* = 2)
UrdA′_R411A_	—	None	46.4 ± 0.04
		100 m*M* phosphate	47.6 ± 0.17
		100 m*M* sulfate	47.2 ± 0.08
	Urocanate	None	46.2 ± 0.12
		100 m*M* phosphate	47.0 ± 0.03 (*n* = 2)
		100 m*M* sulfate	46.7 ± 0.17
	Imidazole propionate	None	46.6 ± 0.23
		100 m*M* phosphate	47.4 ± 0.36
		100 m*M* sulfate	47.3 ± 0.28

## Data Availability

Structure coordinate and data files have been deposited in the Protein Data Bank under PDB codes 9tk1 (UrdA′-RT with sulfate), 9tk3 (UrdA′P-RT with sulfate), 9tk7 (UrdA′P-cryo with sulfate), 9tk5 (UrdA′S-RT), 9tk6 (UrdA′P-RT) and 9tk8 (UrdA′S-cryo).
