# Supplementary material for: Structural insights into urocanate reductase using room-temperature X-ray crystallography
Source: Acta Crystallogr D Struct Biol. 2026 May 5;82(Pt 6):603–14. doi: 10.1107/S2059798326003360 (PMC13224921; doi:10.1107/S2059798326003360)
Supplement: Supplementary file 1 [file d-82-00603-sup1.pdf]

Acta Cryst D
STRUCTURAL
BIOLOGY

Volume 82 (2026)

Supporting information for article:

Structural insights into urocanate reductase using room-temperature X-ray crystallography

Swati Aggarwal, Nitisha Gurav, Esko Oksanen, Karin Lindkvist-Petersson and Raminta Venskutonytė

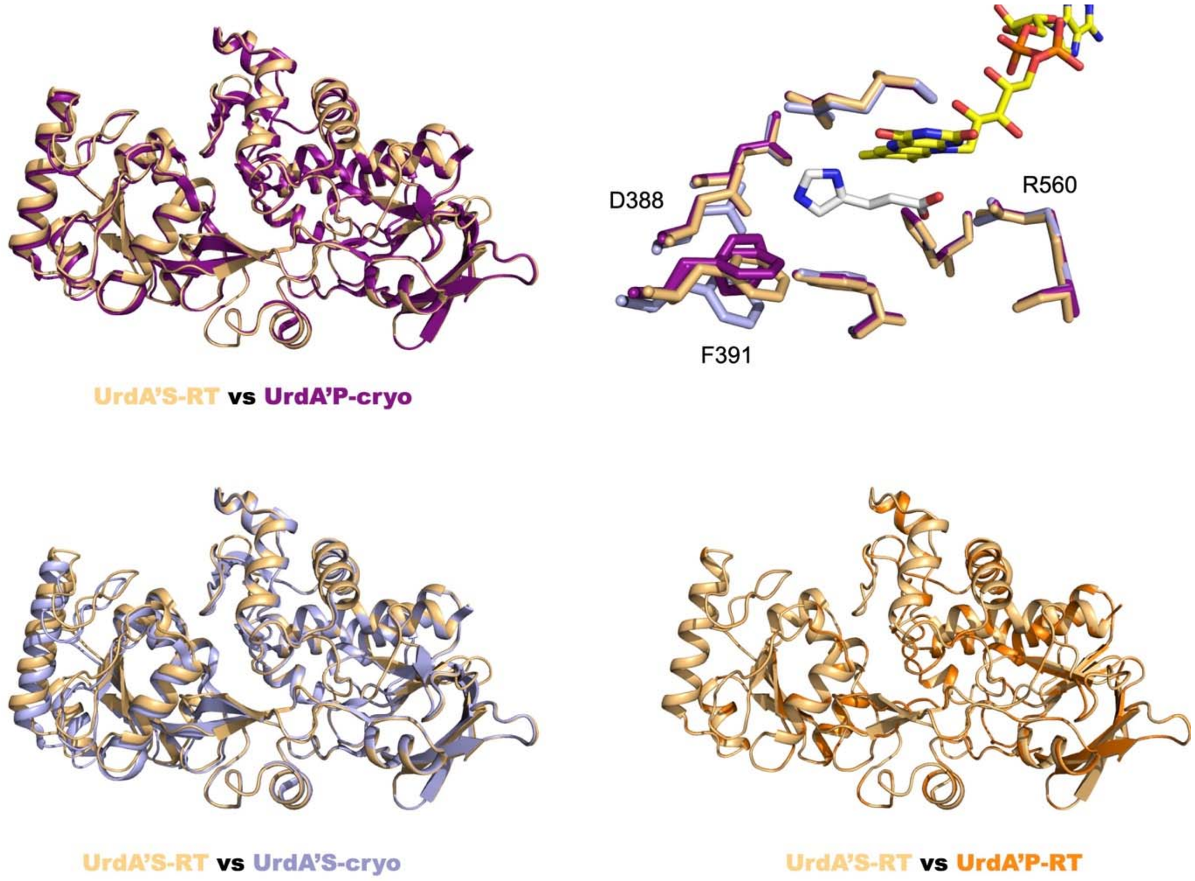

Figure S1 Structure alignments on the FAD domain. Upper panel: UrdA'S-RT structure (beige) overlay with an UrdA'P-cryo (PDB ID 6T88, purple) and a zoom of a binding site of overlayed UrdA'S-RT structure (beige), UrdA'P-cryo (PDB ID 6T88, purple) and UrdA'S-cryo (PDB ID 6T87, light purple); lower panel: UrdA'S-RT structure (beige) overlay with UrdA'S-cryo (PDB ID 6T87, light purple) and UrdA'S-RT (beige) overlay with an UrdA'P-RT (orange).

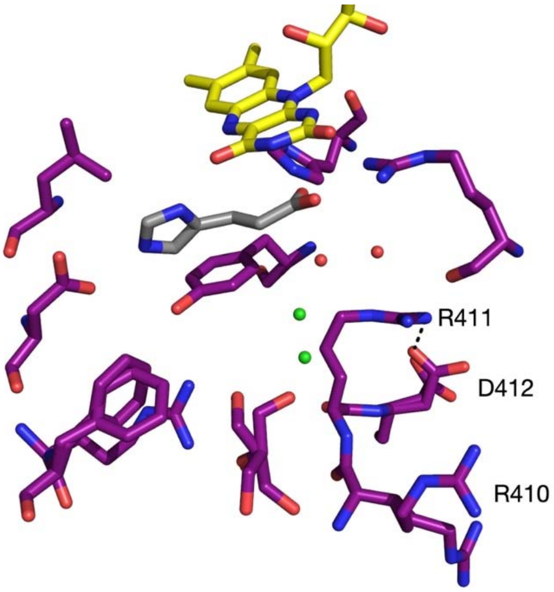

Figure S2 Binding site of the UrdA' in complex with product without sulfate (PDB ID 6T88). Multiple rotamers of Arg410 and Asp412 are present.

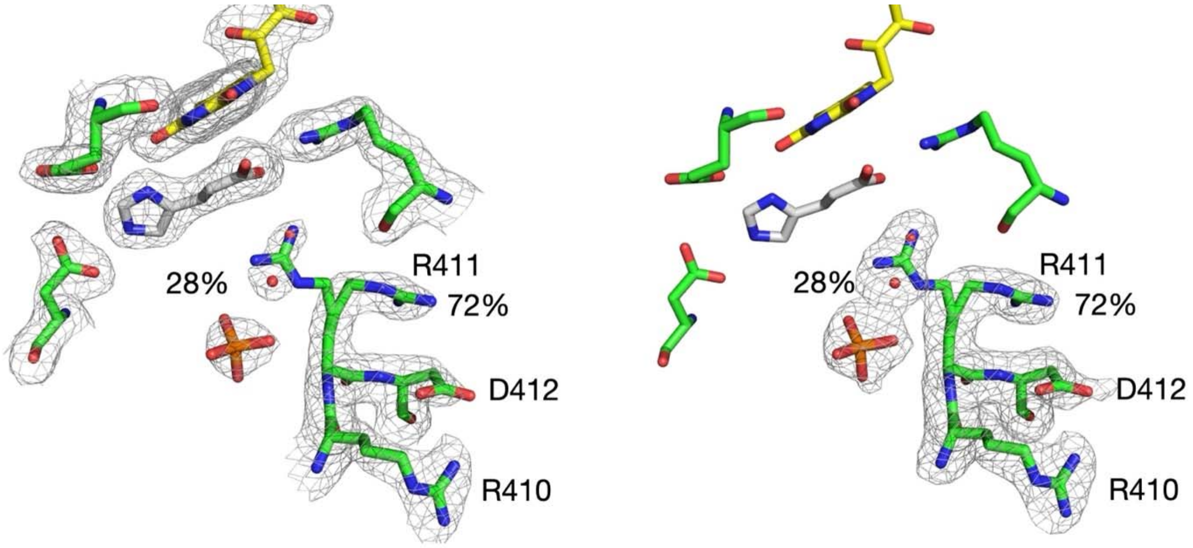

Figure S3 Left: $2m\text{F}_\text{o}$-$D\text{F}_\text{c}$ map shown at 1σ. Right: $m\text{F}_\text{o}$-$D\text{F}_\text{c}$ omit Polder map shown for the residues 410-412 as well as the phosphate and water molecules at 3 σ for the UrdA'S-cryo structure at citrate condition. Occupancies for each of the R411 alternatives are indicated in %.

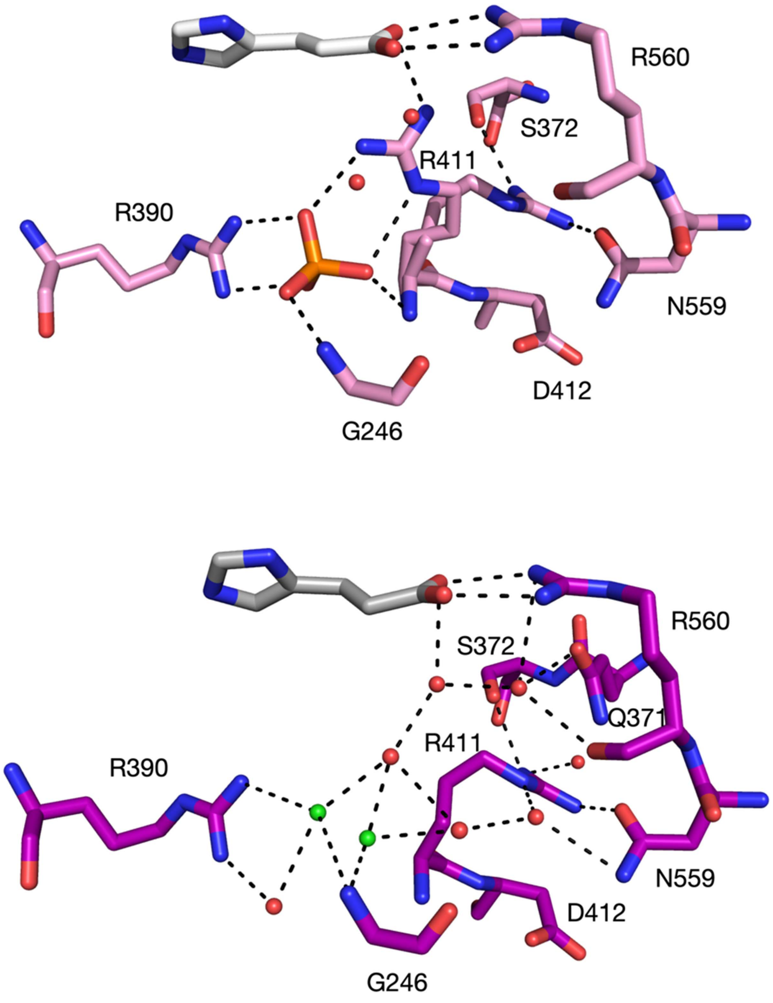

Figure S4 Anion binding site interactions for UrdA'S (top) and UrdA'P (bottom) structures at citrate conditions.
